# Editors’ Pick: A real Caucasian and the genetic investigation of Caucasus peoples

**DOI:** 10.1186/2041-2223-3-15

**Published:** 2012-07-10

**Authors:** Manfred Kayser

**Affiliations:** 1 Department of Forensic Molecular Biology, Erasmus MC University Medical Centre Rotterdam, PO Box 2040, Rotterdam, CA 3000, The Netherlands

## 

**Figure 1:**
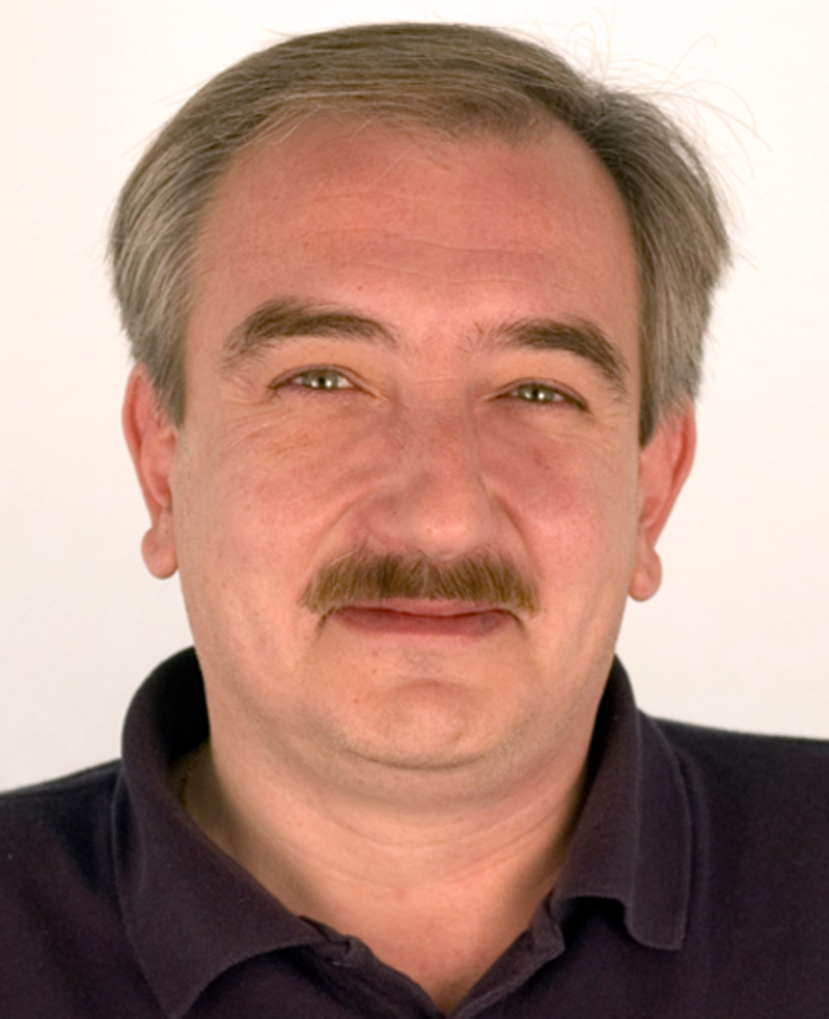
Ivan (Vano) Nasidze, 1964 – 2012. Max Planck Institute for Evolutionary Anthropology.

Referring to Europeans as Caucasians, as still regularly done in the medical and less frequently in the population genetic literature, most often is not justified according to modern scientific views. This term, first mentioned in 1785 by Christoph Meiners, was picked up by Johann Friedrich Blumenbach in his 1795 suggestion of dividing the human world population into 5 groups (Caucasians, Mongolians, Ethiopians, Americans, and Malays). Nevertheless, a Caucasian classification can still be formally correct if it refers to people who originate from the Caucasus region. One real Caucasian, the molecular anthropologist Dr. Ivan (Vano) Nasidze (Figure 1), recently passed away, suddenly and unexpectedly. Although this was a huge shock for everybody who knew Vano, in particular his family and his close colleagues, the way he died seemed the preferred one for many of us similarly passionate scientists who seem unable to live without scientific work. If only, however, it would occur several decades later in life than for Vano who unfortunately did not reach his 48 birthday. He died instantly while working at his office computer at the Department of Evolutionary Genetics, Max Planck Institute for Evolutionary Anthropology Leipzig, Germany.

Ivan Nasidze was a real Caucasian; not only born and raised in Tblisi (Georgia), he dedicated almost all of his professional life to the genetic investigation of human population relationships and history in the Caucasus region. His latest (and now last) in a series of articles on this topic was published in September last year in the *European Journal of Human Genetics*[1]. Using complete mitochondrial (mt) genomes (whereas traditionally only the mtDNA control region - HV1 - is applied) Nasidze and his colleagues found astonishingly high genetic diversity with 144 different sequences and 97 haplogroups among 147 individuals tested. Strikingly, all of the Georgians, Armenians and Iranians (the latter used for comparison) had different mtDNA sequences, while for the Azeri and the Turks (the latter used for comparison) minor within-group haplotype sharing was observed. These data once again underline the extremely high genetic diversity of the Caucasus people as result of a very complex population history. Nasidze and his coauthors confirmed their previous findings that Indo-European-speaking Armenians and Turkic-speaking Azeri are more closely related genetically to Caucasian-speaking groups in the Caucasus than to other Indo-European-speaking and Turkic-speaking groups. The language shift in the Caucasus, which Nasidze’s previous work already had highlighted, was confirmed with this study. However, in contrast to his previous work using solely HV1, these whole mtDNA genome data now indicate some maternal genetic relationship between Turks and Azeri (both Turkic-speaking groups) suggesting that the language shift in the Azeri probably did involve some maternal gene flow from Turks. Hence, applying current next generation sequencing technology to anthropological questions, Nasidze with this work partly confirmed his earlier findings but also revealed new insights into the human genetic history of the Caucasus. Moreover, this study shows, as may be expected, that individuals with identical HV1 sequence are clearly differentiated based on their whole mtDNA genome data, which has significance for anthropological and also forensic studies.

I will miss Vano as a friendly and cooperative colleague who was deeply committed to explore the genetic history of the Caucasus peoples with various molecular approaches (including Y-chromosomal and autosomal DNA variation). I sincerely hope that Dr. Ivan Nasidze will be remembered for his important contributions to the investigative genetics and the molecular anthropology of the Caucasus for a long time to come.
